# Uncertainty Assisted Robust Tuberculosis Identification With Bayesian Convolutional Neural Networks

**DOI:** 10.1109/ACCESS.2020.2970023

**Published:** 2020-01-28

**Authors:** Zain Ul Abideen, Mubeen Ghafoor, Kamran Munir, Madeeha Saqib, Ata Ullah, Tehseen Zia, Syed Ali Tariq, Ghufran Ahmed, Asma Zahra

**Affiliations:** 1 Department of Computer ScienceCOMSATS University Islamabad (CUI)Islamabad44000Pakistan; 2 FET - Computer Science and Creative TechnologiesUniversity of the West of EnglandBristolBS16 1QYU.K.; 3 Department of Computer Information SystemsCollege of Computer Science and Information TechnologyImam Abdulrahman Bin Faisal University 48023 Dammam34212Saudi Arabia; 4 Department of Computer ScienceNational University of Modern Languages (NUML)Islamabad44000Pakistan; 5 Department of Computer ScienceNational University of Computer and Emerging Sciences (NUCES)Karachi54700Pakistan

**Keywords:** Tuberculosis identification, computer-aided diagnostics, medical image analysis, Bayesian convolutional neural networks, model uncertainty

## Abstract

Tuberculosis (TB) is an infectious disease that can lead towards death if left untreated. TB detection involves extraction of complex TB manifestation features such as lung cavity, air space consolidation, endobronchial spread, and pleural effusions from chest x-rays (CXRs). Deep learning based approach named convolutional neural network (CNN) has the ability to learn complex features from CXR images. The main problem is that CNN does not consider uncertainty to classify CXRs using softmax layer. It lacks in presenting the true probability of CXRs by differentiating confusing cases during TB detection. This paper presents the solution for TB identification by using Bayesian-based convolutional neural network (B-CNN). It deals with the uncertain cases that have low discernibility among the TB and non-TB manifested CXRs. The proposed TB identification methodology based on B-CNN is evaluated on two TB benchmark datasets, i.e., Montgomery and Shenzhen. For training and testing of proposed scheme we have utilized Google Colab platform which provides NVidia Tesla K80 with 12 GB of VRAM, single core of 2.3 GHz Xeon Processor, 12 GB RAM and 320 GB of disk. B-CNN achieves 96.42% and 86.46% accuracy on both dataset, respectively as compared to the state-of-the-art machine learning and CNN approaches. Moreover, B-CNN validates its results by filtering the CXRs as confusion cases where the variance of B-CNN predicted outputs is more than a certain threshold. Results prove the supremacy of B-CNN for the identification of TB and non-TB sample CXRs as compared to counterparts in terms of accuracy, variance in the predicted probabilities and model uncertainty.

## Graphical abstract

Bayesian-based convolutional neural network (B-CNN) for tuberculosis identification: Input chest x-ray is given to N number of CNNs. Dropout is enabled at testing stage to get posterior probability distribution (PPD) of classes.

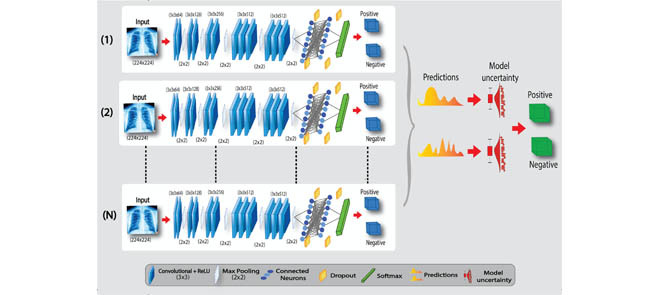

## I. Introduction

Tuberculosis (TB) is a contagious disease that is designated among 10 highest causes of death and also the leading infectious disease above than human immunodeficiency Virus (HIV)/ acquired immune deficiency syndrome (AIDS) [1]. Each year millions of people get infected with tuberculosis. In 2017, approximately 1.3 million deaths were recorded for HIV-negative people. Additionally, the number of deaths among HIV-positive were 3 million. The top estimation was that in total 10 million individuals suffered from TB infection disease in 2017. In which men were 5.8 million, women were 3.2 million and children were 1 million. According to the World Health Organization (WHO), the affected tuberculosis individuals were from all countries and age groups, i.e., over 90% were adults whereas, 9% of people were living with HIV. Furthermore, WHO 2018 TB report listed 30 high TB burden countries from which eight highest TB affected countries were: India (27%), China (9%), Indonesia (8%), Philippines (6%), Pakistan (5%), Nigeria (4%), Bangladesh (4%) and South Africa (3%). The other 22 countries were considered for 87% of the world’s TB infected cases. Only 6% of global cases were in the European and American region (3% each) [1]. Moreover, WHO in its “End-TB Strategy” emphasizes on the timely and accurate diagnosis of TB in patients and recommends the use of chest radiography, or chest X-ray (CXR). While CXR is a commonly utilized tool for diagnosing pulmonary TB, the expertise of radiology interpretation is insufficient in TB dominant areas, which harm the efficacy of triaging and screening of TB [2]. For mass screening, an efficient and low-cost computer-aided solution can be vital for earlier identification of TB disease in developing countries [3]. In mass screening, precise disease identification and evaluation relies on the technologies that require image acquisition and image interpretation. The objective of these technologies is to overcome human interpretation issues such as restricted subjectivity, huge variations among human interpreters, high-cost and limited human resource and fatigue [4]. Such computer-aided solutions are likely to decrease the risk of false detections and facilitate mass screening efforts. These computer-aided solutions can highlight abnormalities and characterizes lung patterns to assist physicians and provide them with a second opinion. The identification of TB from CXRs is a challenging task that requires identification of patterns such as cavity, air space consolidation, endobronchial spread and pleural effusions [5].

**FIGURE 1.**
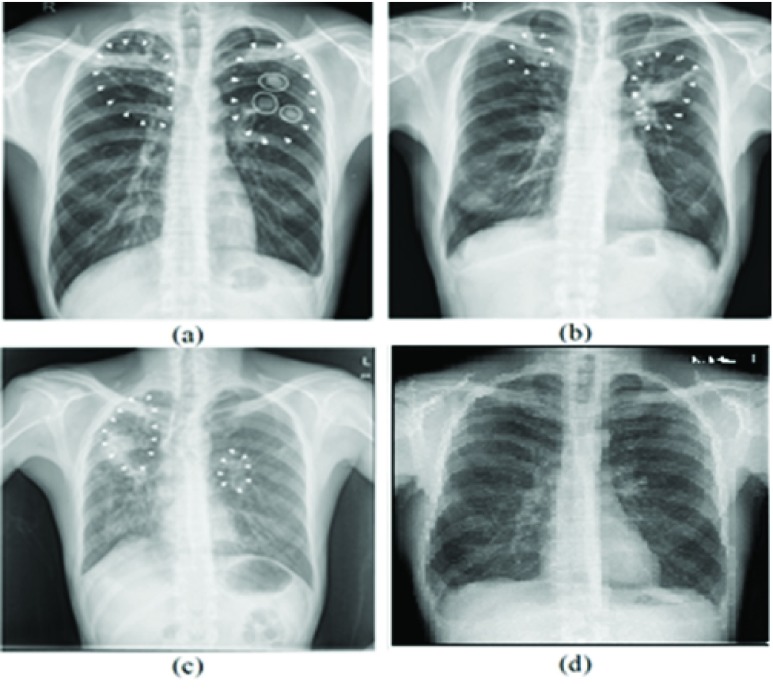
TB manifestation marked with white arrowheads whereas white circles show air space consolidation.

Figure 1(a)-(c) shows sample CXR images where TB manifestation features are visible. White circles in Figure 1(a) represent air space consolidation. In Figures 1(b)-(c), spots and shaded area with ribcage depicts, endobronchial spread and the pleural effusions appearances, which are highlighted with the help of white arrowheads. In contrast, Figure 1(d) shows a normal CXR where lungs do not have any spots or shaded area other than the ribcage. As shown in Figure 1(a)-(c) TB manifested regions are overlapping that makes TB identification a challenging and complex task.

Researchers presented a number of mechanisms to differentiate between the CXRs of TB and non-TB patients. Machine learning (ML) and image analysis techniques are key components behind the recent developments in computer-aided image interpretation systems. Different researchers applied numerous techniques for feature-extraction including Gabor, histogram of oriented gradients (HOG) [6] and speeded up robust features (SURF) [7]. These features are then utilized for TB identification using ML classifiers, such as support vector machine (SVM) and regression tree (CART) [8], [9]. However, these approaches require handcrafted features for classification which do not signify all possible data exemplification.

Deep learning (DL) is an emerging development in ML and image analysis that has been designated as one of the ten breakthrough technologies of this decade. DL is a resurgence of convolutional neural network (CNN) with the capability of learning higher levels of abstraction which is crucial for improving the performance of data analysis algorithms [10]. DL based CNNs are evolving as the foremost ML approach for computer vision [11]. CNN based extracted representations are useful for recognizing and localizing objects in natural images [12]. However, due to the lack of labeled training data CNN model leads towards overfitting [13]. Furthermore, the CNN utilizes the softmax [14] in the last layer to classify the CXR as TB and non-TB. Though, the Softmax tends to classify CXR with a higher probability which is incorrectly interpreted as model confidence. In Bayesian CNN (B-CNN) the dropout between weighted layers is incorporated which can be interpreted as an approximation Bayesian model. The B-CNN for TB diagnostic provides inference ability with confidence.

The main problem is that uncertainty factor is not considered for TB diagnosis in most of the schemes including [15]–[17]. In case of higher values of uncertainty, the decision should be verified by the radiologists. Due to low explicitly and challenging inference of standard TB screening procedures, level of uncertainty may increase [18]. To address this problem, we proposed a novel dropout based Bayesian convolutional neural network which is utilized to robustly identify TB manifested CXRs. The main contribution of our work are as follows;

1) Explored extensive literature to analyze radiograph screen schemes based on machine learning and deep learning algorithms. Through literature study we have identified the problem.
2) Deep learning based TB diagnosis scheme is presented for effective mass screening of CXRs. In the proposed scheme three CNN architectures inspired by VGG-19 are presented to diagnose TB manifestations.
3) Identification of the softmax function utilized at the end of CNNs can only classify the CXR as TB and non-TB with higher confidence which is error prone to the borderline TB cases.
4) B-CNN based model is presented to identify TB by considering the uncertainty factor that effects results. It assigns a higher uncertainty/confusion for erroneous predictions to make better decisions and avoid false positive cases.

Rest of the paper is organized as follows: Sections 2 elaborates the related work and Section 3 explains the DL preliminaries. The proposed TB identification methodology based on B-CNN is presented in Section 4. Furthermore, the results are presented in Section 5 followed by the conclusion.

## II. Related Work

In this Section, we explore a number of TB diagnosis schemes based on machine-learning and deep learning. TB diagnosis relies primarily on radiological patterns. Early detection of TB can lead to its treatment and prevention of infection growth. However, lack of diagnostic resources such as skilled radiologists are major hindrance for effective and robust TB diagnostics. In comparison with human diagnoses, computer-aided tools can provide more significant results with less diagnostic errors and make it possible to perform efficient mass screening with fewer resources.

### A. Machine Learning Based TB Diagnosis

This section describes TB diagnosis schemes based on machine learning (ML) algorithms. The machine learning approaches rely on hand crafted features selection [19] which may work for one scenario but, fail in another. Therefore, ML algorithms demonstrate not to be used full for mass TB screening. Chauhan *et al.* [6] extracted features from CXRs using Gabor filter, gist and variants of HOG like representing shape with a spatial pyramid kernel [20] and Histograms of oriented gradients for human detection [21]. The authors then used SVM [22] for classification and reported accuracy of 94.2% and 86.0% with gist and HOG features, respectively. Alfadhli et al. used SURF [7] for features calculation from varied window sizes used as input for SVM based classifier achieving 89% area under the curve (AUC) [15]. Hogeweg *et al*. presented a feature extraction and classification mechanism to compute local pixel characteristics. The method uses position features, texture features and features derived from the Hessian matrix. The hybrid active shape model pixel algorithm was used for classification. The receiver operator characteristic (ROC) based accuracy of 84.7% was achieved [8]. Jaeger et al. presented a TB diagnostic scheme. The authors have experimented two features, i.e., features of object detection as set A, and features that are based on image retrieval as set B. furthermore, the set A comprises on histogram for intensity, gradient, magnitude, shape-descriptors, curvature-descriptors, HoG and LBP. The set B comprises on descriptor such as Tamura-texture, edge and color, fuzzy-color, color-layout [23]. In [24], Yahiaoui et al. presented preliminary diagnosis of TB by using SVM for TB and non-TB manifestations. It involves private dataset for training and testing. The ML techniques for TB identification have achieved good results. However, these technique fails to address the uncertain cases due to features selection by human experts which are hand-crafted that may work for some certain scenarios and fail for others situations. On the other hand, DL based techniques are most suitable as the features and representations are extracted automatically from image data by using the pattern learning and back-propagation.

### B. Deep Learning Based TB Diagnosis

This section presents TB diagnostics methods based on Deep learning (DL). The DL-based methods achieved significant results for computer aided diagnostics. Lopes and Valiati [25] proposed bags-of-features and ensemble-based method to extract the features from segmented CXRs. The authors have collected features by using multiple CNNs including Visual Geometry Group (VGG) [26], Residual neural network (ResNet) [27] and GoogleNet [16]. For the collected features, the authors trained SVM for classification. The classification achieved AUC 78.2%, 74.6%, and 75.3% on GoogleNet, ResNet, and VggNet, respectively. Lakhani et al. presented an ensemble of AlexNet and GoogleNet and attained AUC of 99%. It uses two non-publically available datasets with unbalanced classes which can effect generalized efficacy of TB identification [3]. In [9], Évora et al. developed Artificial neural networks (ANN) to diagnose drug-resistant TB (DR-TB) for their own collected dataset. The dataset consisted of 280 subjects and achieved a classification accuracy of 88.1%. Dataset is not publically available for experiments.

Alcantara et al. extracted features with GoogleNet and performed classification using SVM on their own collected dataset which comprised of 4701 images. It achieved 89.6% accuracy for binary classification of TB and 62.07% accuracy for multi-class classification of different TB manifestations. However, classes in dataset are uneven as Lymphadenopathy has 202 images whereas Infiltration comprises of 2252 images [28]. In [29], Melendez et al. explored three techniques including SVM, multi-instance learning (MIL) and active learning (AL) for TB diagnosis. It uses a dataset of 917 CXRs including 392 normal and 525 TB cases. In this scenario, SVM achieved highest AUC of 90%. Vajda *et al.* [30] presented TB diagnosis using segmentation based on deep learning. Authors achieved 95.6% accuracy with AUC of 99% on Shenzhen dataset. The method performs segmentation on lung images. After segmentation, authors have extracted shape descriptor histogram of lung shape descriptor. For classification of TB manifests, authors have utilized a simple neural network in [31] that achieved 90.3% accuracy and 96.4% AUC. It used transfer-learning mechanism with ImageNet where 10848 CXRs were used for training purpose. In [32], Islam et al. have presented a methodology which is based on AlexNet, VGG and ResNet models. ResNet achieved higher accuracy in comparison to AlexNet and VGG, i.e., 88% and 86.2% respectively. However, the ResNet achieved 91% AUC, which is lower than the other state-of-the-art. Authors also presented an ensemble comprises of six CNN models and achieved 90% accuracy with AUC of 0.94. In [23], a CNN-based TB diagnose scheme is presented that uses mimic AexNet network architecture. For training, Shenzen datasets were used. Although, the scheme achieves accuracy of 84.4% but probability of classification from DL techniques generally inferred as model confidence that is not true for all scenarios. Conventional DL methods for identification and regression are not enough capable of detecting the model uncertainty.

In our proposed work, we focus on recent model uncertainty integration tools with DL algorithm to cope with uncertain CXRs. In uncertain CXRs, it is difficult to identify whether TB manifestations are present or not in confusing cases. Softmax tends to misclassify TB from CXRs and represents model confidence either very high or very low which is not true. The next section explains the detailed technical background of DL approaches used for TB identification.

## III. Preliminaries of Deep Learning

DL techniques can learn the TB manifestation patterns and features automatically from raw CXR data used for classification and localization task. These DL techniques are capable of learning features at different abstraction levels by piling non-linear layers that allow the DL techniques to attain better simplification for difficult computer vision tasks such as image localization, segmentation, and classification.

### A. Convolutional Neural Networks

CNN is a DL based approach that became an emerging technique for image classification due to its significant achievements. The success achieved is because of the precise and robust assumptions of CNN for the natural image data including associations and pixel locality [26]. Secondly, CNN optimizes the tasks by significantly less number of parameters as compared to feed-forward networks [12]. For classification tasks, layers preserve features and patterns that are essential for identification and throwaway irrelevant variations. Basic architecture of CNN consists of following layers.

#### 1) Convolution Layer

The units of convolution layer are linked to their respective units of the local patch coming from the preceding layer with a filter. The units are activated by using the feature map, which is computed through applying Rectified Linear Unit (ReLU) [33] above the locally weighted sums.

#### 2) Pooling Layer

This layer works by merging features and patterns semantically into a solitary feature map. The pooling layer computes the maximum or average of input features from the previous layer and uses them as an output feature map.

#### 3) Fully-Connected Layer

In the fully-connected layer, every unit is connected to previous layer units and making a mesh. Usually, before fully-connected layer two or three stacks of the convolution and pooling layers are placed to extract features.

#### 4) Softmax Layer

The purpose of the softmax layer is to converts the features into probabilities which belong to each output class. The total number of units in the softmax layer is equal to the total output classes. The softmax function is defined by equation (1) where Softmax $\left ({\textrm {a}_{\textrm {i}} }\right)$ and ${a}_{i}$ represent the feature and the probability of ${i}^{th} $ class respectively. The ${e}^{a}_{i}$ is the non-normalized probability measurement and $\sum \nolimits _{j=1}^{m} {e}^{a}_{j}$ is used for normalizing the distribution of probability over $m$ output classes [14]:

\begin{equation*} Softmax\left ({a_{i} }\right)= \frac {e^{a_{i}}}{\sum \nolimits _{j=1}^{m} e^{a_{j}}}\tag{1}\end{equation*}

ReLU can be used as an activation function for displaying non-linearity as well as achieving faster learning convergence [33]. Learning phase deals with weight optimization of the units to minimize erroneous classifications. Optimizer such as stochastic gradient descent is typically used to gradients for all the weights which are computed with the back-propagation algorithm.

### B. Bayesian Convolutional Neural Networks

To cope with low visibility between CXRs, a model is needed that should be proficient enough to represent the prediction uncertainty. The state-of-the-art techniques such as [34], [35] and [36], are kernel-based methods where pairs of images are checked for similarity measurement. The measured similarity is fed to the classifier as input such as in SVM. However, the proposed methodology focuses on utilizing the efficacy of CNN and highlight the utility of Bayesian uncertainties approximation [37]. The preceding probability distributions of B-CNN are defined for a set of parameters, such as ${\omega =\left \{{{Wht}_{1}, {\ldots }, {Wht}_{L}}\right \}}$ where ${Wht}_{L}$ are the weights of ${L}^{th}$ layer. A probability model can be described by a standard Gaussian assumption over ${\omega }$. Probability of class ${y}$ equals to ${c}$ given input $\mathsf {x}$ is defined by using softmax [14] function. It includes parameter weights ${\omega }$ for feature set ${f}$ as given in Equation (2).

\begin{equation*} p\left ({{y=c}\thinspace \vert \thinspace {\mathsf {x}, \omega }}\right) =softmax\left ({f^{\omega }\left ({\mathsf {x} }\right) }\right)\tag{2}\end{equation*}

The inference in B-CNN model is implemented by using the initial stochastic regularization method such as dropout [38], [13]. The Dropout is added after every fully-connected and convolution layers in B-CNN model. During the testing phase and approximate posterior sampling, dropout is also employed. This is equal to carrying out an estimated variational inference which aims to find a manageable distribution ${q}_{\theta }^{\ast }{(\omega)}$ using a dataset for training $\mathcal {D}_{train}$. It is accomplished by reducing Kullback-Leibler (KL) divergence with model posterior ${p}\left ({{\omega }\thinspace \vert \thinspace \mathcal {D}_{{\textrm {train}}}}\right) {.}$ The dropout layer helps to maintain uncertainty in weights during prediction through relegating the estimated posterior via employing integration of Monte Carlo as given in equation (3) [39] where ${q}_{\theta }^{\ast }(\omega)$ is referred to as dropout distribution [40] and $\hat {\omega }$ represents the sample weights from the distribution. Equation (4) averages output probability with respect to $\hat {\omega }$ for the ${T}$ number of stochastic forward passes. The vital characteristics of B-CNN for this work is its efficiency to deal with small datasets [37] and possessing of uncertainty information to deal with the uncertain cases [40].

\begin{align*} p\left ({{y=c}\thinspace \vert \thinspace {\mathsf {x}, \mathcal D_{train}}}\right)=&\int {p\left ({{y=c}\thinspace \vert \thinspace {\mathsf {x}, \omega }}\right)} \\ p\left ({\omega \thinspace \vert \thinspace \mathcal D_{train}}\right) d\omega{\approx }&\int {\mathrm {p}\left ({{\mathrm {y=c}}\thinspace \vert \thinspace {\mathsf {x, \omega }}}\right)} \mathrm {q}_{\theta }^{\ast }\left ({\omega }\right)\mathrm {d\omega } \tag{3}\\{\approx }&\frac {1}{{\textrm {T}}}\sum \nolimits _{{\textrm {t}=1}}^{\textrm {T}} {{\textrm {p}}\left ({{{\textrm {y}=\textrm {c}}}\thinspace \vert \thinspace {\mathsf {x,}\hat {\omega }_{\textrm {t}}}}\right)}\tag{4}\end{align*}

By exploring the literature, we have identified that the state-of-the-art schemes are CNN-based and uses softmax for classification of TB and non-TB. The use of Softmax at the end of CNN architecture make the inference either 0 or 1 which means the CNN will always confidently predict between ‘Yes’ or ‘No’. Due to softmax classifier, the state-of-the-art schemes either cannot identify reliably or inference is not correctly predicted. To address this issue we have deployed a Bayesian base CNN architecture which robustly infer the TB and non-TB manifestations.

## IV. Proposed Methodology

In this Section, a robust TB diagnostic scheme based on Bayesian is presented. We have explored three baseline-CNN architectures that are trained on two benchmark datasets named Montgomery and Shenzhen datasets. Moreover, we have deployed Bayesian based CNN (B-CNN) architecture to overcome the softmax inference issue. We have calculated variance from prediction results of B-CNN on Shenzhen dataset to validate the robustness of proposed B-CNN. List of notations is presented in TABLE 1.

**TABLE 1:** List of Notations

Sr.	Notation	Description
1.	$x$	Input defined by using softmax
2.	$\mathrm {D\_{}train}$	Training dataset
3.	${\mathrm {q}}\_{}\theta ^{*(\omega)}$	Dropout distribution
4.	T	Number of stochastic forward passes
5.	I = {$\text{I}_{\mathrm {1..}}\text{I}_{\mathrm {N}}$}	Set of CXRs
6.	Y = {$\text{y}_{\mathrm {1..}}\text{Y}_{\mathrm {N}}$}	Set of parallel labels
7.	i	Convolutional layer
8.	$\text{t}\times \text{t}$	Size of convolution layer
9.	$\text{b}_{\mathrm {i}}$	Variational parameters
10.	$\text{M}_{\mathrm {i}}$	Bernoulli distribution
11.	$\text{p}_{\mathrm {i}}$	Dropout probabilities
12.	$\upsilon $	Variance
13.	$\text{k}^{\mathrm {((r))}}$	Extraction of features component
14.	$\text{z}^{\mathrm {((r))}}$	Extracts features
15.	${\mathrm {bias}}^{\left ({r }\right)}$	Biases at $\text{r}^{\mathrm {th}}$ layer

By applying B-CNN, TB identification task can be described as follows; consider a set of CXRs denoted as $I =\left \{{ {I}_{1}{,}{I}_{2}{,\ldots,}{I}_{N}}\right \}$, where each CXR is described as CXR ${I \in }\,\,{{[{0; 1}]}}^{m \times n}$ (${m}$ is the height and ${n}$ is the width of all the CXRs denoted by ${N}$ number of CXRs) as a set of parallel labels $Y =\left \{{ {y}_{1}{,}{y}_{2}{,\ldots,}{y}_{N}}\right \}$ where all the labels corresponds to a binary classification result ${U \in [{0; 1}]}$. The function ${f}$ learns to map the input CXR ${I}$ to equivalent labels ${Y}$. The output label presented by ${U}_{out}$ which is equal to the ground-truth label represented by ${U}_{\textrm {gt}}$. The B-CNN architecture of proposed TB identification methodology is based on dropout [39], which is utilized for variational inference in B-CNN [37]. Authors in [37], have described a relation between variational inference and dropout for B-CNN by Bernoulli distributions and CNN weights. This similar approach is used to represent uncertainties for the B-CNN while CXRs classification. In this proposed study, the network weights of B-CNN are utilized to learn the posterior distribution. The CXR training data ${I}$ and labels ${Y}$ are given as ${p}\left ({{\omega }\thinspace \vert \thinspace {I,Y}}\right) $. In general, this distribution is not tractable; therefore, the distribution over the weights is requisite for approximation [37]. The variational inference is employed for approximating the weights [37]. This process helps to improve the distribution approximated over weights ${q(\omega)}$, through reducing the KL divergence among ${q}\left ({{\omega } }\right)$ and ${p(\omega \vert I,Y)}$ as given by equation (5) where ${q}\left ({{\omega }_{i} }\right)$ is defined for each convolutional layer ${i}$ of ${t\times t}$ size, having ${j}$ units. Furthermore, ${b}_{i,j}{\sim Bernoulli}\left ({{p}_{i} }\right)$ for $\text {j}=1, 2,\ldots, \text {t}_{i}$ and ${\omega }_{i}={M}_{i}{\textrm {diag}}\left ({{b}_{i} }\right)$. In this scenario, ${b}_{i}$ and ${M}_{i}$ are vectors for variables distribution with variational parameters and Bernoulli distribution, respectively. Although, the optimization for dropout probabilities ${p}_{i}$, are at a standard value of 0.5 [13].

\begin{equation*} KL\left ({q (\omega)\vert \big | p \left ({\omega \vert I,Y}\right)}\right)\tag{5}\end{equation*}

In [37], the authors describe that, in order to reduce KL divergence, they minimized the cross-entropy loss function. Hence, using stochastic gradient descent for network learning acquires a network weights distribution. The B-CNN model is trained by adding dropout for the CXRs classification. To obtain the class probabilities posterior distribution over the weights, the dropout is also added at testing-phase. The variance $\boldsymbol {\upsilon }$ and mean of the samples are utilized as uncertainty and confidence, for the CXRs classification respectively. A simple heuristic function for confusion is ${if~\upsilon \ge \tau }$ whereas in case of no-confusion ${if~\upsilon < \tau }$ defines the confusion while prediction where ${\tau }$ is threshold. If the variance of B-CNN prediction is above the ${\tau }$, it would mean that classifier is uncertain or confused about TB existence in the CXR and the CXR should be further analyzed by an expert radiologist for the final decision.

**FIGURE 2.**
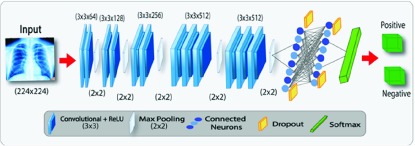
Schematic diagram of the proposed TB identification.

Proposed CNN architecture for CXRs classification is shown in Figure 2. Primarily, it comprises of three components including extraction of features, selection of features, and prediction. All component contains a sequence of procedure which comprises on layer functionality. The extraction of features component at stage ${k}^{\left ({{r} }\right)}$ extracts features ${Z}^{\left ({{r} }\right)}$ as given by the equation (6) where ${\ast }$ operator denotes convolution, ${\omega }^{(r)}$ is weights and ${bias}^{(r)}$ is biases at the ${r}^{th}$ layer. ${Z}^{(r-1)}$ is the input CXR ${I}$ for $r = 1$ where ${Z(0) = I}$ as ${r-1}^{th}$ hidden layer activation for ${r > 1}$.

\begin{align*}&\hspace {-0.5pc}{Z}^{\left ({{r} }\right)}{=}{k}^{\left ({{r} }\right)}\left ({{Z}^{\left ({{r-1} }\right)}{; }{\omega }^{\left ({{r} }\right)}{, }{bias}^{\left ({{r} }\right)} }\right){normalize} \\&\qquad \qquad\quad\quad {{\displaystyle {\left ({{pool}\left ({{relu}\left ({{\omega ^{\left ({{r} }\right)}\,\,{\ast \,\,Z}}^{\left ({{r-1} }\right)}{+}\,\,{bias}^{\left ({{r} }\right)} }\right) }\right) }\right)} }}\tag{6}\end{align*}

Moreover, it includes the computation of operations such as non-linear transformation, convolution, local normalization and max-pooling [12]. The feature selection component at stage ${f}^{\left ({{r} }\right)}$ is expressed as given in equation (7) where ${(.)}$ specifies dot product as it involves dot product operation. Furthermore, ${Z}^{(r-1)}$ is ${r-1}^{th}$ activation for the hidden layer. Precisely, it includes dot product operation tailed by non-linear transformation.

\begin{align*} {Z}^{\left ({{r} }\right)}=&{f}^{\left ({{r} }\right)}\left ({{Z}^{\left ({{r-1} }\right)};{ }{\omega }^{\left ({{r} }\right)},{ }{bias}^{\left ({{r} }\right)} }\right) \\=&\left ({{\text {relu}}\left ({{\omega ^{\left ({{r} }\right)}.{Z}}^{\left ({{r-1} }\right)}+{bias}^{\left ({{r} }\right)} }\right) }\right)\tag{7}\end{align*}

Lastly, prediction component involves the softmax [14] function which provides the probability ${p}$ for each neuron indicating the possible class as output ${C}$. It can be formulated as equation (8).

\begin{equation*} {p}\left ({{C}\thinspace \vert \thinspace {I;\omega, bias}}\right) {=softmax}\left ({{\omega ^{\left ({{r} }\right)}.~Z}^{\left ({{r-1} }\right)} + {bias}^{\left ({{r} }\right)} }\right)\tag{8}\end{equation*}

To construct TB identification based on B-CNN model, the three components are stacked as in equation (9):

\begin{align*} {p}\left ({{C}\thinspace \vert \thinspace {I; \omega, bias}}\right)=&softmax \\=&\Bigg (\!{\text {f}}^{\left ({{5} }\right)}\!\left ({{\text {f}}^{\left ({{4} }\right)}\!\left ({\! {\text {k}}^{\left ({{3} }\right)}\left ({{\text {k}}^{\left ({{2} }\right)}\left ({{\text {k}}^{\left ({{1} }\right)}\left ({{\text {I}} }\right) }\right) }\right) \!}\right) \!}\right) \!\!\Bigg)\tag{9}\end{align*}

Features are extracted and selected by CNN components. Additionally, ReLU is utilized as the nonlinearity activation function [40]. Next, we explore the three baseline CNN architectures as illustrated in TABLE 2. These are used to compare the efficiency of CNN.

**TABLE 2:** Description of the Baseline CNN Architectures Used to Explore the Efficacy of CNN

Architecture – 1			Architecture – 2		Architecture – 3	
Layer #	Layer type – size	Activation Function	Layer type – size	Activation Function	Layer type – size	Activation Function
1	Convol-1 – $\mathrm {3 \times 3\,\times 64}$	ReLU	Convol-1 – $\mathrm {3 \times 3\,\times 64}$	ReLU	Convol-1 – $\mathrm {3 \times 3\,\times 64}$	ReLU
2	Convol-2 – $\mathrm {3 \times 3\,\times 64}$	ReLU	Convol-2 – $\mathrm {3 \times 3\,\times 64}$	ReLU	Convol-2 – $\mathrm {3 \times 3\,\times 64}$	ReLU
3	Pooling-1 – $\mathrm {2 \times 2}$	–	Pooling-1 – $\mathrm {2 \times 2}$	–	Pooling-1 – $\mathrm {2 \times 2}$	–
4	Convol-3 – $\mathrm {3 \times 3\, \times 128}$	ReLU	Convol-3 – $\mathrm {3 \times 3\, \times 128}$	ReLU	Convol-3 – $\mathrm {3 \times 3\, \times 128}$	ReLU
5	Convol-4 – $\mathrm {3 \times 3\, \times 128}$	ReLU	Convol-4 – $\mathrm {3 \times 3\, \times 128}$	ReLU	Convol-4 – $\mathrm {3 \times 3\, \times 128}$	ReLU
6	Pooling-2 – $\mathrm {2 \times 2}$	–	Pooling-2 – $\mathrm {2 \times 2}$	–	Pooling-2 – $\mathrm {2 \times 2}$	–
7	Convol-5 – $\mathrm {3 \times 3\, \times 256}$	ReLU	Convol-5 – $\mathrm {3 \times 3\, \times 256}$	ReLU	Convol-5 – $\mathrm {3 \times 3\, \times 256}$	ReLU
8	Convol-6 – $\mathrm {3 \times 3\, \times 256}$	ReLU	Convol-6 – $\mathrm {3 \times 3\, \times 256}$	ReLU	Convol-6 – $\mathrm {3 \times 3\, \times 256}$	ReLU
9	Pooling-3 – $\mathrm {2 \times 2}$	–	Pooling-3 – $\mathrm {2 \times 2}$	–	Convol-7 – $\mathrm {3 \times 3\, \times 256}$	ReLU
10	Convol-7 – $\mathrm {3 \times 3\, \times 512}$	ReLU	Convol-7 – $\mathrm {3 \times 3\, \times 512}$	ReLU	Pooling-3 – $\mathrm {2 \times 2}$	–
11	Convol-8 – $\mathrm {3 \times 3\, \times 512}$	ReLU	Convol-8 – $\mathrm {3 \times 3\, \times 512}$	ReLU	Convol-8 – $\mathrm {3 \times 3\, \times 512}$	ReLU
12	Convol-9 – $\mathrm {3 \times 3\, \times 512}$	ReLU	Convol-9 – $\mathrm {3 \times 3\, \times 512}$	ReLU	Convol-9 – $\mathrm {3 \times 3\, \times 512}$	ReLU
13	Pooling-4 – $\mathrm {2 \times 2}$	–	Pooling-4 – $\mathrm {2 \times 2}$	–	Convol-10 – $\mathrm {3 \times 3\, \times 512}$	ReLU
14	Convol-10 – $\mathrm {3 \times 3\, \times 512}$	ReLU	Convol-10 – $\mathrm {3 \times 3\, \times 512}$	ReLU	Pooling-4 – $\mathrm {2 \times 2}$	–
15	Convol-11 – $\mathrm {3 \times 3\, \times 512}$	ReLU	Convol-11 – $\mathrm {3 \times 3\, \times 512}$	ReLU	Convol-11 – $\mathrm {3 \times 3\, \times 512}$	ReLU
16	Pooling-5 – $\mathrm {2 \times 2}$	–	Convol-12 – $\mathrm {3 \times 3\, \times 512}$	ReLU	Convol-12 – $\mathrm {3 \times 3\, \times 512}$	ReLU
17	FC-1 – 4096	ReLU+Drop	Pooling-5 – $\mathrm {2 \times 2}$	–	Convol-13 – $\mathrm {3 \times 3\, \times 512}$	ReLU
18	FC-2 – 4096	ReLU+Drop	FC-1 – 4096	ReLU+Drop	Pooling-5 – $\mathrm {2 \times 2}$	–
19	FC-3	Softmax	FC-2 – 4096	ReLU+Drop	FC-1 – 4096	ReLU+Drop
20	–	–	FC- 3	Softmax	FC-2 – 4096	ReLU+Drop
					FC-3	Softmax

* Conv.: Convolutional, D: Dropout, FC: Fully Connected

**FIGURE 3.**
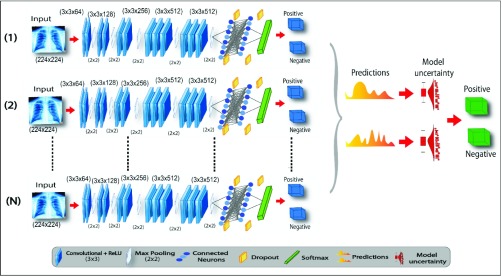
Illustration of the proposed TB identification based on B-CNN Testing method.

**FIGURE 4.**
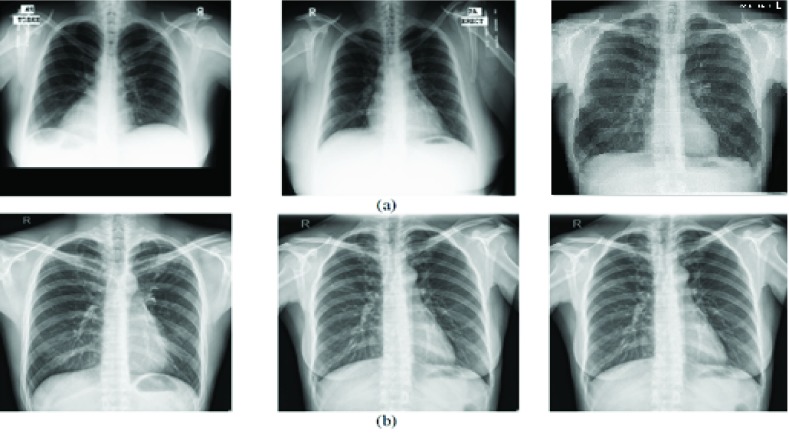
Sample CXRs from both datasets. (a) Montgomery. (b) Shenzen.

Figure 3 illustrates overall functionality of the proposed TB identification methodology based on B-CNN model. The model comprises of ${1, 2, \ldots, N}$ layers where each layer involves baseline-CNN architecture-2. The input CXR is processed by all these layers of B-CNN to further perform simultaneous prediction. In this model, we enable the dropout at the time of prediction which in result produces different probabilities for classification. However, the magnitude of variation between the probabilities of CXR inference shows either the model is predicting with high/low confidence which shows the model uncertainty. If the variation is high then, the model uncertainty is also high which demonstrates that the model has low confidence while predicting CXR. In case of low uncertainty, the mean of predicted probabilities from B-CNN model is considered as final prediction of CXR. In Figure 4, CXRs are passed to $N$ number of CNN models that generate $N$ outputs. Mean of these outputs reflect confidence of B-CNN model with final classification result. However, each CNN produces different outputs due to addition of dropout at testing-phase. The uncertainty or confusion of B-CNN model is estimated by extracting variance values from the output of $N$ number of CNN models.

## V. Results and Discussion

In this section, we explore experimental setup and B-CNN model based on baseline-CNN architectures. We analyze baseline-CNN architectures conversion into B-CNN and their prediction probability. In the last, we have discussed in detail important part of model uncertainty and its role to pronounce the model confidence. In this study, two benchmark datasets including Montgomery and Shenzhen [23] are used for evaluating the efficacy of proposed TB identification methodology. Shenzhen dataset consists of 662 grayscale CXRs where 336 are positive TB CXRs and 326 are negative TB CXRs. The Montgomery dataset consists of 138 grayscale CXRs, where 58 CXRs are TB positive, and 80 CXRs are non-TB. Figure 4(a) and Figure 4(b) shows sample images from Montgomery and Shenzhen, respectively. For training, 80% of the both datasets are used, while the other 20% of each dataset is used for the testing. The experiments are performed on three different baseline CNN architectures as shown in Table 1. All the training and the testing experiments are performed on the platform provided by Google Colab with 16 GB of RAM and NVidia Tesla K80 GPU with ~12 GB VRAM [41].

### A. Accuracy of TB Identification

The identification accuracy is used to gauge the performance of proposed TB identification methodology based on B-CNN in comparison with other techniques from literature including: SVM [15], AlexNet [12], VGG16 [26], VGG19 [26], ResNet [42], and CNN based methodologies proposed by Lopes and Valiati [25] and Sivaramakrishnan *et al.* [17]. For the experimentation and comparison, three baseline CNN architectures are proposed, which are presented in Table 2 including Architecture-1, Architecture-2, and Architecture-3. The baseline CNN architectures are trained and tested separately for both datasets including Montgomery and Shenzhen [23]. Moreover, the results from Table 3 clearly depicts that all of the proposed CNN architectures outperformed the state-of-the-art DL approaches from the literature in terms of identification accuracies in case of Montgomery. For both datasets, the Architecture-2 shows highest identification accuracy as compared to state-of-the-art DL approaches along with Architecture-1 and Architecture 3.

**TABLE 3:** Result Summary for Both Benchmark Datasets

Method		Identification Accuracy (%)	
		dataset Montgomery	dataset Shenzhen
SVM [15]		79.1	–
CNN [25]		82	84
CNN [17]		75.8	85.5
AlexNet [12]		71.42	84.21
VGG16 [26]		75	81.20
VGG19 [26]		78.57	84.21
ResNet [45]		82.14	83.45
Baseline – CNN	Arc-1	89.47	78.57
	Arc-2	92.85	85.71
	Arc-3	90.22	82.14
Proposed B-CNN	Arc-1	90.22	82.14
	Arc-2	96.42	86.46
	Arc-3	92.85	85.71

**FIGURE 5.**
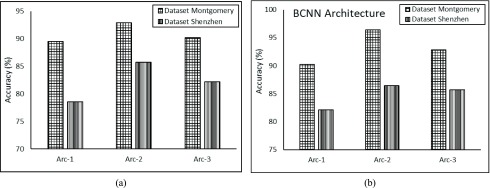
Comparison of (a) baseline- CNN architecture and (b) B-CNN for Montgomery and Shenzhen datasets.

Figure 5(a) elucidates a graphical representation of achieved accuracy for all three proposed baseline-CNN architectures. The baseline-CNN architectures are trained and tested for both Montgomery and Shenzhen datasets. It can be observed that the proposed baseline-CNNs including Arc-1, Arc-2 and Arc-3 correctly predicted TB manifestation and achieved 89.47%, 92.85% and 90.22% test-time prediction accuracy respectively for Montgomery dataset. For Shenzhen dataset, the baseline-CNN Arc-1, Arc-2 and Arc-3 achieved 78.57%, 78.57% and 82.14% test-time prediction accuracy respectively. The test-time accuracy results clearly depict that proposed baseline-CNN arc-2 outperforms other two proposed baseline CNNs.

Figure 5(b) demonstrates results for Bayesian–based CNNs architectures. The test-time prediction accuracy of all three architectures, i.e., Arc-1, Arc-2 and Arc-3 is increased after incorporating Bayesian. The improved prediction accuracy of all architectures is 90.22%, 96.42%, 92.85% respectively for Montgomery. The accuracy of all three B-CNN for Shenzhen dataset is also improved such as Arch-1 achieved 82.14%, Arch-2 achieved 86.46% and, Arch-3 achieved 85.71% test- time prediction accuracies respectively. The results show that Arch-2 B-CNN dominates over other two architecture of B-CNN.

**FIGURE 6.**
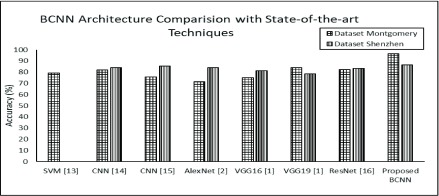
Comparison of baseline- CNN architecture for Montgomery and Shenzhen datasets.

To evaluate the robustness of our proposed scheme we have compared the results of Acr-2 B-CNN with other state-of-the-art schemes. From Figure 6 it can be observed that the proposed Arch-2 B-CNN model accurately predicted the TB manifestations with test-time prediction accuracy 96.42% for Montgomery and 86.46 % for Shenzhen respectively. Whereas for the same experimental settings the other state-of-the-art schemes such as SVM, pertained–CNN1, pertained–CNN2, AlexNet, VGG16, VGG19 and ResNet achieved 79.1%, 82%, 75.8%, 71.42%, 75%, 78.57%, 82.14% respectively for Montgomery dataset. The prediction accuracy results for Shenzhen dataset are 84%, 85.5%, 84.21%, 81.20%, 84.21%, 83.45% respectively for pertained–CNN1, pertained–CNN2, AlexNet, VGG16, VGG19 and ResNet. Result show that proposed Acr-2 B-CNN model performs better as compared to state-of-the-art schemes.

**FIGURE 7.**
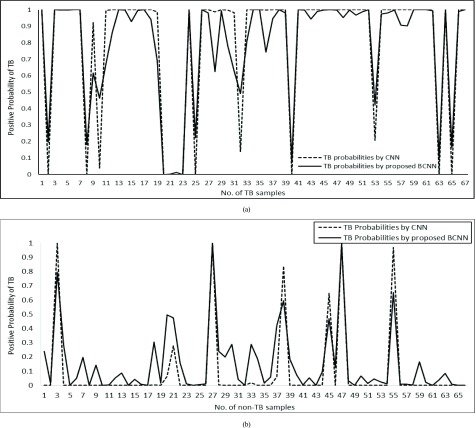
(a) Positive probability of TB in TB manifested CXRs samples. (b) Positive probability of TB in non-TB manifested CXRs samples.

Furthermore, Figure 7(a-b) shows the predictive probabilities of softmax and confidence of B-CNN respectively for TB and Non-TB cases from the Shenzhen dataset. It can be observed from both graphs that softmax tends to be at the extreme in most of the cases which shows its unrealistic behavior. On the contrary, B-CNN provides realistically looking confidence since various examples may vary in confidence regarding their association with classes.

**FIGURE 8.**
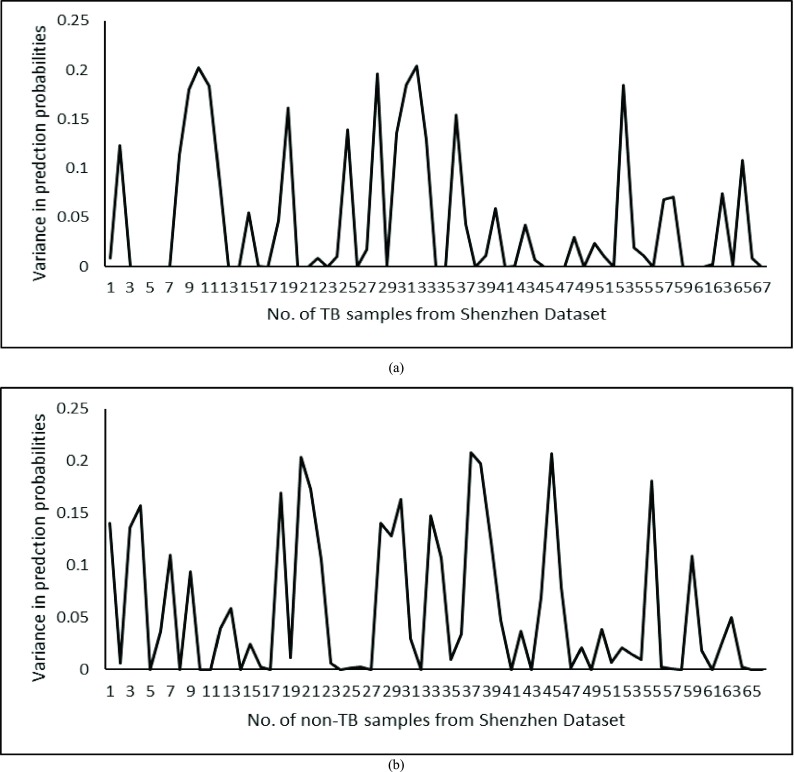
(a) Uncertainty captured while predicting test samples from Shenzhen in TB samples. (b) The captured uncertainty in non-TB samples.

**FIGURE 9.**
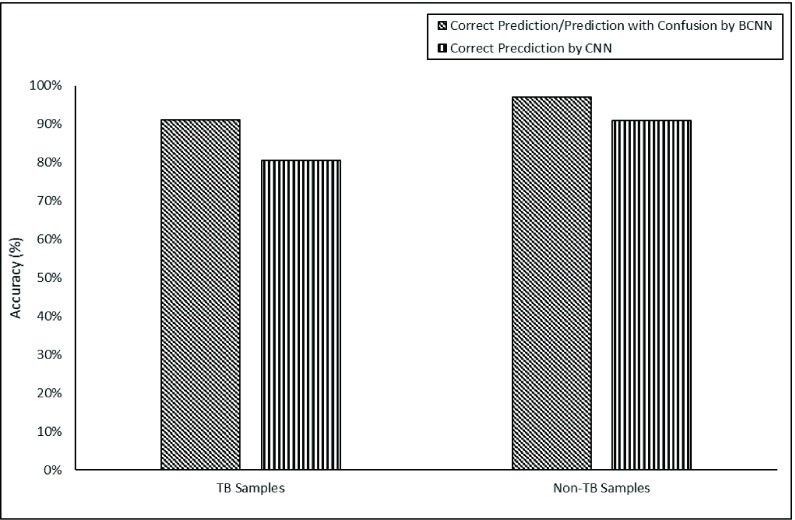
CNN and B-CNN comparison result after threshold on variance while predicting TB and non-TB samples.

**FIGURE 10.**
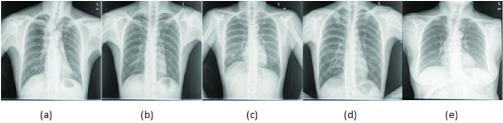
Comparison of sample CXRs from Shenzhen dataset predicted by CNN and the proposed methodology based on B-CNN.

Figure 8 (a) and (b) show variance in probability of TB and non-TB samples by B-CNN. After applying a threshold of 0.1 for TB and non-TB samples from Shenzhen dataset the variance is up to 10.4% of the false negatives (wrong prediction of TB CXRs) and 4.5% of the false positives (wrong prediction of non-TB CXRs) are declared as confusion cases from Shenzhen dataset, respectively. The CXRs which are declared as confusion cases are wrongly predicted by CNN architectures. However, the B-CNN model has either correctly classified those CXRs or has assign them as confusion cases that gives a chance to an expert radiologist for making the final decision. In Figure 9, we further investigated the utility of B-CNN. we have compared the model variance to filter confusion cases after applying threshold on all the TB and non-TB samples from Shenzhen dataset. The prediction accuracy achieved by CNN for TB-samples 80.6%, whereas B-CNN achieved prediction accuracy of 91.04%. For non-TB sample, baseline-CNN achieved prediction accuracy of 90.91% in-comparison to B-CCN achieved prediction accuracy of 96.97%. Proposed TB identification methodology based on B-CNN not only improves the accuracy of TB identification from the CXRs but also validates its prediction. Furthermore, B-CNN can correctly classify up to 93.9% that is either the CXR belongs to TB or non-TB status or is a marginal confusion case that needs to be examined by expert radiologists. Results show that CNN Arc-2 achieves 85.7% accuracy whereas proposed B-CNN achieves 93.9% accuracy using Shenzhen dataset. The effectiveness of B-CNN over baseline CNN models can be further analyzed by Figure 10 that shows the TB prediction results on Shenzhen dataset. Figure 10 (a)-(c) display sample non-TB CXRs. Figure 10 (d) and (e) demonstrate sample TB.

The CXR in Figure 10 (a) is incorrectly predicted as positive for TB by baseline CNN model. However, the proposed TB identification methodology based on B-CNN model correctly predicted it as a non-TB sample. The baseline CNN architectures and B-CNN model wrongly predicted the CXRs in Figure 10(b) to Figure 10 (e); however, the B-CNN model lowered the prediction confidence and showed confusion on those CXRs in comparison to baseline CNN architectures. Moreover, the variance in the prediction of CXRs in Figure 10 (b) to Figure 10 (e) is more than 0.1. Thus by applying a threshold on the variance of predicted CXRs the wrongly predicted CXRs fall into the confusion cases. This proposed TB identification methodology based on B-CNN is significant and novel as it characterizes the wrongly predicted CXRs as confusion cases which can be further analyzed by an expert radiologist. The diagnostic of contagious diseases is crucial through computer-aided solutions as mentioned in the future work of DSW Ting *et al.* [18], there is a need to validate the results of diagnostic. In our work, the proposed B-CNN based TB identification validates its results by utilizing the uncertainty. It shows higher uncertainty where the model faces confusion or wrongly predicts marginal cases among the TB and non-TB manifestations. However, the state-of-the-art DL approaches tend to wrongly predict the borderline TB and non-TB manifestation cases with higher confidence which leads to erroneous prediction. It shows that rather than making classification decisions on model confidence only, the inclusion of uncertainty can improve the decision process.

## VI. Conclusion

TB identification is a challenging task due to the occurrence of complex and obscured patterns present in CXRs. Numerous TB identification approaches are proposed in the literature. However, the accuracy of TB identification is still low. Recently, DL based approaches have presented significant results for TB identification by discovering complex features from large datasets. However, conventional deep learning based models are not capable to suggest uncertainty for output class prediction. Proposed B-CNN exploits the model uncertainty and Bayesian confidence to improve the accuracy of TB identification as well as validation of the results. The dropout is utilized at the training and testing phase to acquire the posterior probability distribution of the classes. The variance in probabilities is utilized as uncertainty and the mean of the posterior probability distribution of the classes is used to make the final output classification decision. The proposed methodology based on B-CNN has experimented on two TB benchmark datasets: Montgomery and Shenzhen. The results have demonstrated that the proposed methodology achieved highly significant results in terms of TB identification accuracy, i.e., 96.42% and 86.46% for both datasets (i.e., Montgomery and Shenzhen) in comparison with the state-of-the-art DL approaches in the literature. Furthermore, by utilizing the variance of proposed TB identification methodology based on B-CNN a threshold of 0.1 is applied to filter out the confusion cases of the Shenzhen dataset. The applied threshold helped to capture the 10.4% of false negative and the 4.5% of false positive as confusion cases. These confusion cases improve the classification in terms of validation up to 93.9% that is either the CXR belongs to TB or non-TB status or is a marginal confusion case that needs to be examined by an expert radiologist. This proposed work shows compelling results and provides an improved foundation for future work. However, the real challenge is to determine that, how such DL systems will accurately fit with the workflow for diagnostic of thorax diseases and clinical screening settings. In future, we shall focus on potential validation that will provide a robust approach to deal with multiple thorax diseases from CXRs. Furthermore, BCNN models shall be utilized to eliminate uncertainty factor in natural images classification.
